# The infectious salmon anemia virus esterase prunes erythrocyte surfaces in infected Atlantic salmon and exposes terminal sialic acids to lectin recognition

**DOI:** 10.3389/fimmu.2023.1158077

**Published:** 2023-04-25

**Authors:** Johanna Hol Fosse, Adriana Magalhaes Santos Andresen, Frieda Betty Ploss, Simon Chioma Weli, Inger Austrheim Heffernan, Subash Sapkota, Krister Lundgård, Raoul Valentin Kuiper, Anita Solhaug, Knut Falk

**Affiliations:** Norwegian Veterinary Institute, Ås, Norway

**Keywords:** receptor destroying enzyme (RDE), *Orthomyxoviridae*, infectious salmon anemia (ISA) virus, red blood cells (erythrocytes), sialic acid, *Salmo salar* (L.)

## Abstract

Many sialic acid-binding viruses express a receptor-destroying enzyme (RDE) that removes the virus-targeted receptor and limits viral interactions with the host cell surface. Despite a growing appreciation of how the viral RDE promotes viral fitness, little is known about its direct effects on the host. Infectious salmon anemia virus (ISAV) attaches to 4-*O*-acetylated sialic acids on Atlantic salmon epithelial, endothelial, and red blood cell surfaces. ISAV receptor binding and destruction are effectuated by the same molecule, the haemagglutinin esterase (HE). We recently discovered a global loss of vascular 4-*O*-acetylated sialic acids in ISAV-infected fish. The loss correlated with the expression of viral proteins, giving rise to the hypothesis that it was mediated by the HE. Here, we report that the ISAV receptor is also progressively lost from circulating erythrocytes in infected fish. Furthermore, salmon erythrocytes exposed to ISAV *ex vivo* lost their capacity to bind new ISAV particles. The loss of ISAV binding was not associated with receptor saturation. Moreover, upon loss of the ISAV receptor, erythrocyte surfaces became more available to the lectin wheat germ agglutinin, suggesting a potential to alter interactions with endogenous lectins of similar specificity. The pruning of erythrocyte surfaces was inhibited by an antibody that prevented ISAV attachment. Furthermore, recombinant HE, but not an esterase-silenced mutant, was sufficient to induce the observed surface modulation. This links the ISAV-induced erythrocyte modulation to the hydrolytic activity of the HE and shows that the observed effects are not mediated by endogenous esterases. Our findings are the first to directly link a viral RDE to extensive cell surface modulation in infected individuals. This raises the questions of whether other sialic acid-binding viruses that express RDEs affect host cells to a similar extent, and if such RDE-mediated cell surface modulation influences host biological functions with relevance to viral disease.

## Introduction

Sialic acids are highly diverse (>80 derivatives known to date) and typically present on the outermost ends of glycans attached to plasma membrane-anchored proteins or lipids (1). Many sialic acid-binding viruses express a receptor-destroying enzyme (RDE) that removes the virus-targeted receptor and limits viral host cell attachment (2). An appropriate balance between viral receptor-binding and receptor-destroying activities promotes viral fitness. The RDE supports both early and late steps in the infectious cycle: First, RDE activity destroys decoy receptors in mucus and reduces the cell surface density of virus-targeted sialic acids; this appears to help virus particles reach sites on the plasma membrane that favor viral entry (3–6). Second, when new virus particles bud from the plasma membrane, RDE activity is required to prevent aggregation of viral particles and allow their release (7). The importance of viral receptor destruction can be exemplified by the strong reduction of influenza virus replication by compounds that inhibit its RDE, the neuraminidase (6). In addition, viral receptor destruction mediates attachment interference that limits host cell superinfection (8–11).

Despite the growing appreciation of the role of the viral RDE in the infectious cycle, little is known about its direct effects on the host. First, how extensive is the RDE-mediated modulation of target cell surfaces in an infected individual? Second, considering that cell surface sialic acids modulate a range of cellular functions, including the activation of immune responses (12–14), could the loss of sialic acid viral receptors influence host biological functions with relevance to viral disease?

Infectious salmon anemia virus (*Isavirus salaris*, ISAV) is an enveloped, segmented, single-stranded, negative sense RNA virus of the *Orthomyxoviridae* family. ISAV contains eight genomic segments that encode at least 10 proteins (15–17). Amongst these is the dual-function surface glycoprotein haemagglutinin esterase (HE) that is responsible for both binding to and hydrolysis of 4-*O*-acetylated sialic acids, identified as the ISAV receptor (18, 19). Infection with pathogenic ISAV variants causes disease (infectious salmon anemia, ISA) in farmed Atlantic salmon (*Salmo salar* L.) (20), and has led to vast economic losses in all major salmon-producing countries.

Vascular endothelial cells are the main target cells of pathogenic ISAV and support the generation of new virus particles that are released into the blood stream (21, 22). Moreover, clinical signs of ISA are compatible with a viral sepsis-like breakdown of central vascular functions, including petechial bleeds, vascular leakage, and focal necrosis (20). We recently revealed a global loss of vascular 4-*O*-acetylated sialic acids in ISAV-infected fish that correlated with the expression of viral proteins (23). While this suggested that the esterase activity of the HE could be involved in the host cell surface modulation, we could not exclude the involvement of endogenous host esterases.

Several lines of reasoning made us curious if erythrocyte surfaces in infected fish were modulated in a similar manner: First, nucleated fish erythrocytes are targeted by ISAV, but show limited, if any, permissiveness to infection (24). Consequently, any surface modulation would be independent of the cellular expression of viral proteins. Second, prior to the onset of sepsis-like clinical signs and mortality, ISAV-infected fish typically develop anemia (24, 25). In general, sialic acids and their 9-*O*-acetylation are involved in regulating the circulating half-life of erythroid-lineage cells in other species (26–29); hence, a modulation of erythrocyte sialic acids could potentially contribute to the pathogenesis of ISA. Finally, erythrocytes are accessible to sampling and *ex vivo* manipulation, facilitating exploration of underlying mechanisms.

We found that erythrocytes in infected fish, similar to endothelial cells, progressively lost the ability to bind new ISAV particles. By exposing erythrocytes from non-infected fish to ISAV and recombinant proteins *ex vivo*, we further revealed that the loss of ISAV binding was not due to saturation of the receptor, but associated with pruning of surface sialic acids by the ISAV esterase. These findings expand on our recent observation of loss of vascular 4-*O*-acetylated sialic acids in infected fish, as we provide direct mechanistic evidence that the surface modulation is caused by ISAV esterase activity, rather than endogenous host esterases. Moreover, we demonstrate that cells can be extensively modulated by RDE activity despite not permitting viral replication. Our observations raise the questions of whether RDEs of other sialic acid-binding viruses modulate target cell populations to the same extent, and if such surface modulations influence biological processes in infected hosts.

## Results

### Disease and viraemia in experimentally infected fish

**Figure 1:**
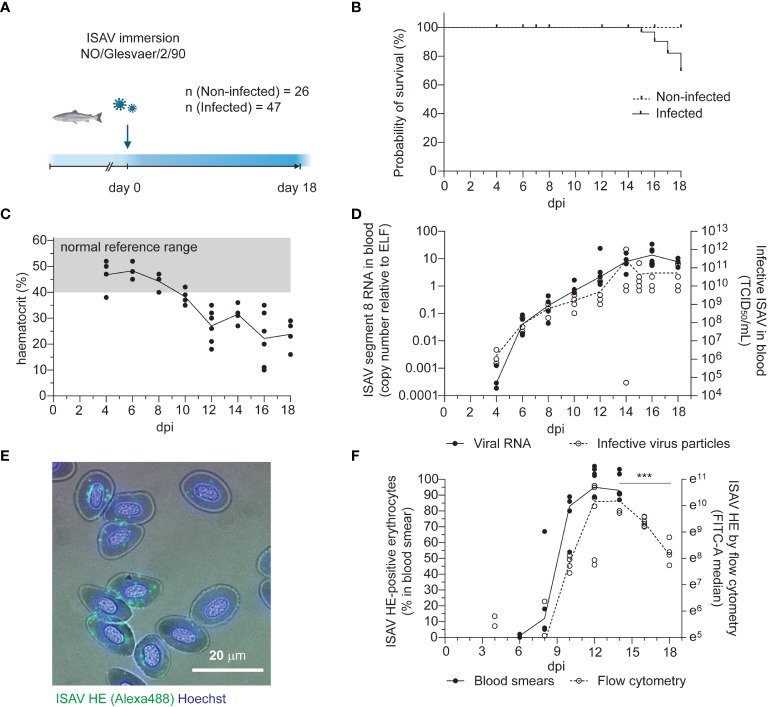
Disease and viraemia in experimentally infected fish. **(A)** Outline of the infection trial. Atlantic salmon were challenged with ISAV (NO/Glesvaer/2/90, 2 hours immersion) and followed for 18 days. The illustration was created in BioRender.com. **(B)** Cumulative mortality in the infected fish group. No deaths occurred in the non-infected group. **(C)** Hematocrits (HCT) of infected fish. Data points represent individual fish, and the line connects median values. The reference range (grey shading) is based on blood samples from 25 non-infected fish (mean +/- 2 standard deviation). **(D)** Viral RNA and infective particles in blood were measured by qPCR targeting ISAV segment 8 (left y-axis, black dots and line) and titration on ASK cells (right y-axis, open circles, stippled line). Data points represent individual fish, lines connect median values. **(E)** Representative micrograph of an acetone-fixed blood smear from an infected fish (12 dpi) immunostained for HE (clone 3H6F8, green/Alexa488). Nuclei are counterstained with Hoechst 33342 (blue), and bright field contrast shows the outline of the cells. The scale bar measures 20 μm. **(F)** The percentage of HE-positive cells in acetone-fixed blood smears were counted manually (black dots and line). PFA-fixed blood cells from infected fish were immunostained for HE, and the signal was measured by flow cytometry (open circles, stippled line). Data points represent individual fish. Lines connects median values. ***p<0.001: flow cytometry data from 14, 16, and 18 dpi were compared by Kruskall-Wallis test and Dunn’s multiple comparison (**Table S1**).

Atlantic salmon (n = 47, median body weight 115 g) were challenged by immersion for two hours with ISAV (Glesvaer/2/90, 10^3.75^ TCID_50_/mL) and subsequently maintained in a fresh water flow-through system at 12°C for the duration of the trial (**Figure 1A**). The first mortality in the infected fish group occurred 15 days post infection (dpi), with mortality rapidly increasing over the next days (**Figure 1B**). The trial was terminated 18 dpi, upon reaching its pre-determined end point of 40% cumulative mortality. No fish in the non-infected group (n = 26) died during the trial. Infected fish developed anemia 12 dpi (**Figure 1C**), prior to other clinical signs. Histological examination of hematoxylin & eosin-stained sections of formalin-fixed paraffin-embedded tissues revealed increased erythrophagocytosis in head kidney and spleen of infected fish that coincided with the onset of anemia (**Figures S1A–C**), suggesting that erythrocytes were removed from the circulation at an increased rate. Viraemia, measured by ISAV segment 8 RNA and infective particles in the blood, was detected in 3 out of 4 fish at the earliest sampling point (4 dpi) and all infected fish at subsequent samplings. Blood viral loads peaked and plateaued 14 dpi (**Figure 1D**), with no significant decline within the trial period (**Table S1**). Levels of viral RNA in the head kidney peaked and plateaued earlier, 10 dpi (**Figure S1D**), in line with the assumption that circulating ISAV particles originate from extensive replication in vascular endothelial cells (21, 22).

Erythrocyte-associated ISAV was first evaluated by manual counting of immunostained blood smears, detecting ISAV-positive erythrocytes in 2 out of 4 fish 6 dpi and all fish at subsequent samplings (**Figures 1E, F**, black dots). Blood smears obtained at 16 and 18 dpi were not of sufficient technical quality for analysis. Flow cytometry provided a more objective method for quantifying the erythrocyte-associated ISAV, and was performed at day 4, 8, 10, 12, 14, 16, and 18 dpi (**Figure 1F**, open circles, stippled line, **Figure S1E**). A decline in ISAV-positive cells was detected from 14 to 18 dpi (**Figure 1F** and **Table S1**). Both the percentage of ISAV-positive cells assessed by immunostaining of blood smears (Spearman r = 0.8302, p < 0.001, **Table S1**) and the flow cytometry-measured ISAV HE signal (Spearman r = 0.6774, p < 0.0001, **Table S1**) correlated with infective titers.

### The ISAV receptor is lost from circulating erythrocytes in infected fish

**Figure 2:**
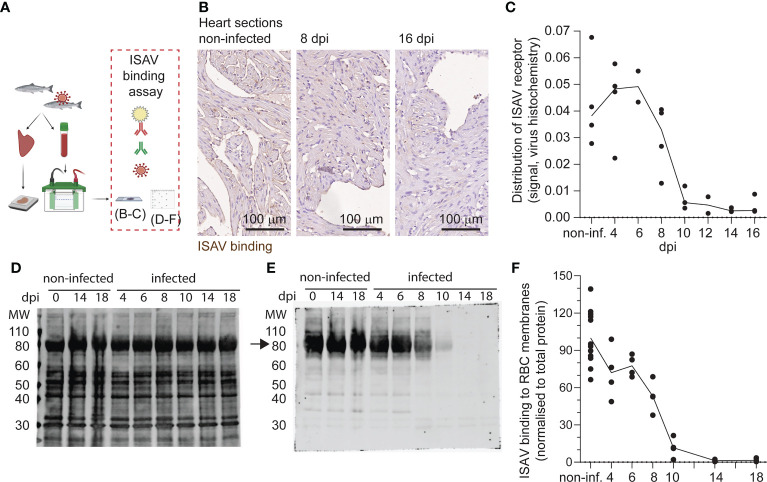
Like vascular endothelium, circulating erythrocytes in infected fish lose the ability to bind new ISAV. **(A)** The distribution of the ISAV receptor in heart tissues and membrane-enriched erythrocyte fractions was evaluated by serial incubation with ISAV antigen, mouse IgG_1_ targeting HE (clone 3H6F8), fluorescence- or HRP-conjugated secondary antibodies, and substrate if relevant. The illustration was created in Biorender.com. **(B)** Representative micrographs showing the ISAV receptor (brown) in hearts of non-infected and infected fish (8 and 16 dpi). Sections are counterstained with hematoxylin. Scale bars measure 100 μm. **(C)** Quantification of ISAV receptor signal in scanned heart sections. **(D, E)** Membrane-enriched erythrocyte lysates were separated by SDS-PAGE and blotted to nitrocellulose membranes, before evaluation of ISAV binding as described above. Representative blots show **(D)** total protein and **(E)** ISAV binding (arrow) to samples from non-infected fish harvested 0, 14, and 18 dpi and infected fish harvested 4, 6, 8, 10, 14, and 18 dpi. **(F)** Quantification of signal (4 fish per time point) normalized to total protein. **(C, F)** Data points represent individual fish. Lines connect median values.

Next, we assessed the distribution of the ISAV receptor in tissue sections and membrane-enriched fractions of erythrocytes from infected fish. We performed a virus binding assay where ISAV antigen produced in infected cells was used as the primary probe (21) (**Figure 2A**). Consistent with our recent observations (23), heart vascular endothelial cells lost their capacity to bind new ISAV particles 10 dpi and did not recover (**Figures 2B, C**). Despite ISAV remaining attached to circulating erythrocytes throughout the course of infection (**Figure 1F**), a similar loss of the capacity to bind new ISAV particles was observed 10 dpi onwards (**Figures 2D–F**). New ISAV binding to heart sections and erythrocyte membranes correlated in individual fish (Spearman’s correlation coefficient = 0.7494, p < 0.0001, **Table S1**). These findings reveal that erythrocytes in ISAV-infected fish lose the ability to bind new ISAV particles as the infection progresses in a similar manner to vascular endothelial cells (23), suggesting loss of the host cell surface receptor.

### The erythrocyte loss of ISAV binding is not mediated by saturation of the ISAV receptor

**Figure 3:**
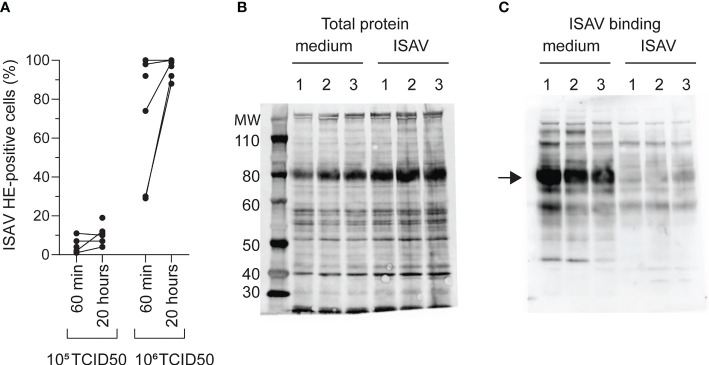
Loss of ISAV binding is not mediated by saturation of its cellular receptor. **(A)** Density-purified erythrocytes isolated from non-infected fish were incubated with ISAV at the indicated dose (per 20 million cells) and given duration, and the percentage of HE-positive cells was quantified by flow cytometry. Data points show measurements in cells from individual fish, with lines connecting values from the same fish when relevant. **(B, C)** Membrane-enriched erythrocyte lysates from three fish (1, 2, 3) exposed to medium or ISAV (10^5^ TCID_50_ per 20 million cells, 20 hours) were separated by SDS-PAGE and blotted to nitrocellulose membranes. **(B)** Blots were stained for total protein by Revert700 Total Protein Stain, **(C)** and the level of ISAV receptor was evaluated by serial incubation with ISAV antigen, mouse IgG_1_ targeting HE (clone 3H6F8), HRP-conjugated secondary antibody, and substrate. The assay was repeated twice with identical results. The arrow points to the band representing ISAV binding in medium-treated erythrocytes.

To understand the mechanisms behind the observed loss of the ISAV receptor in infected fish, we next exposed density-purified erythrocytes from non-infected fish to supernatants from ISAV-infected cells. After identifying a virus dose that did not saturate the ISAV binding capacity (10^5^ TCID_50_ per 2 × 10^7^ cells, **Figure 3A**), we evaluated ISAV binding in cells exposed to this non-saturating dose for 20 hours. Reflecting the situation in infected fish, we found that prior exposure to ISAV abolished subsequent ISAV binding to the membrane-enriched fractions (**Figures 3B, C**).

This illustrates that exposure to ISAV-containing supernatants is sufficient to reproduce the loss of ISAV binding observed in infected fish, and that the loss of binding is not due to receptor saturation.

### The loss of ISAV binding is accompanied by increased availability to sialic acid-binding lectins

Loss of 9-*O*-acetylation can make sialic acids more available to endogenous sialic acid-binding immunoglobulin-like lectins (siglecs), probably by reducing steric hindrance (29–31). Similarly, 4-*O*-acetylation prevents binding of the plant lectin wheat germ agglutinin (WGA) to a range of α2,3-linked sialic acids (32). Four members of the siglec family (siglec 1, 2, 4, and 15) are conserved in all vertebrates, including Atlantic salmon (33). As the binding specificities of Atlantic salmon siglecs have not been characterized, we here used WGA as a proxy for testing if the loss of the ISAV receptor could modulate interactions between lectins and Atlantic salmon erythrocytes.

**Figure 4:**
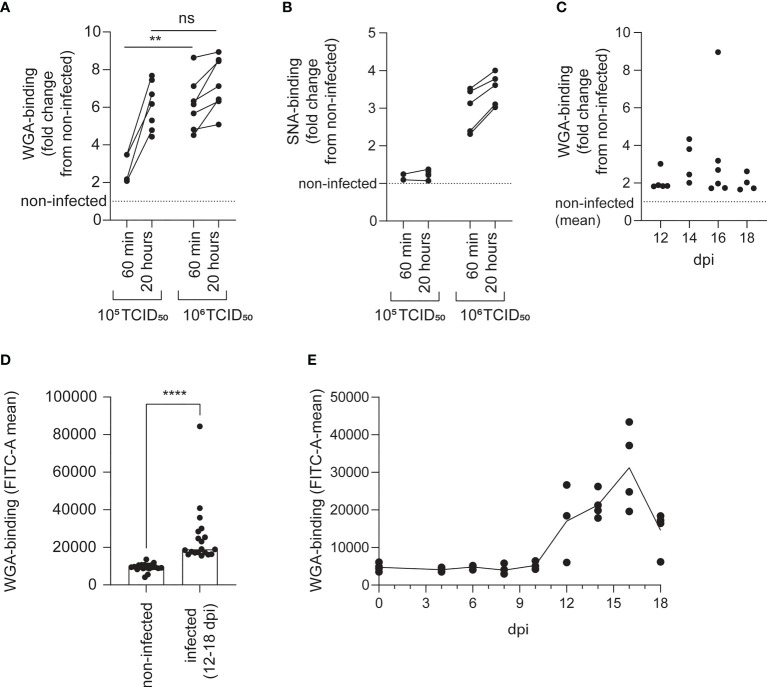
The loss of ISAV binding is accompanied by increased availability to sialic acid-binding lectins. The lectin-binding capacity of erythrocytes was evaluated by flow cytometry. **(A, B)** Density-purified erythrocytes isolated from non-infected fish were incubated with ISAV at the indicated dose (per 20 million cells) and given duration, and WGA-binding **(A)** and SNA-binding **(B)** was quantified in live cells by flow cytometry. Data points show measurements in cells from individual fish, with lines connecting values from the same fish. ** p < 0.01, Mann-Whitney U, ns, non-significant. **(C, D)** WGA-binding to PFA-fixed blood cells from infected fish was quantified by flow cytometry. Data points show values in individual fish. **** p < 0.0001, Mann-Whitney U. **(E)** Density-purified erythrocytes were incubated with plasma from infected fish harvested at the indicated time points (10 µL/10^6^ cells) and incubated overnight before quantification of WGA-binding by flow cytometry. Data points show cells incubated with plasma from individual fish (4 per time point). The line connects medians.

We found that exposure to both low (10^5^ TCID_50_ per 2 × 10^7^ cells) and high (10^6^ TCID_50_ per 2 × 10^7^ cells) doses of ISAV increased the binding of WGA to erythrocytes (**Figure 4A**). The effect was evident as soon as 60 min after exposure. At this time, the low-dose effect was less prominent, but after 20 hours, the low and high doses of virus increased WGA binding to a similar extent (**Figure 4A** and **Table S1**). Only the high dose of ISAV increased binding of the sambucus nigra lectin (SNA), which specifically targets α2,6-linked sialic acids (32) (**Figure 4B**).

Erythrocytes of infected fish (12-18 dpi) also bound WGA more efficiently than cells from non-infected individuals harvested at the same time (**Figures 4C, D**), confirming the *in vivo* relevance of our finding. Finally, incubation of erythrocytes from healthy fish with plasma from infected fish increased their binding to WGA (**Figure 4E**). Due to the limited amount of sample available, SNA-binding was not tested in samples from infected fish.

### The modulation of erythrocyte surfaces is mediated by the ISAV esterase

**Figure 5:**
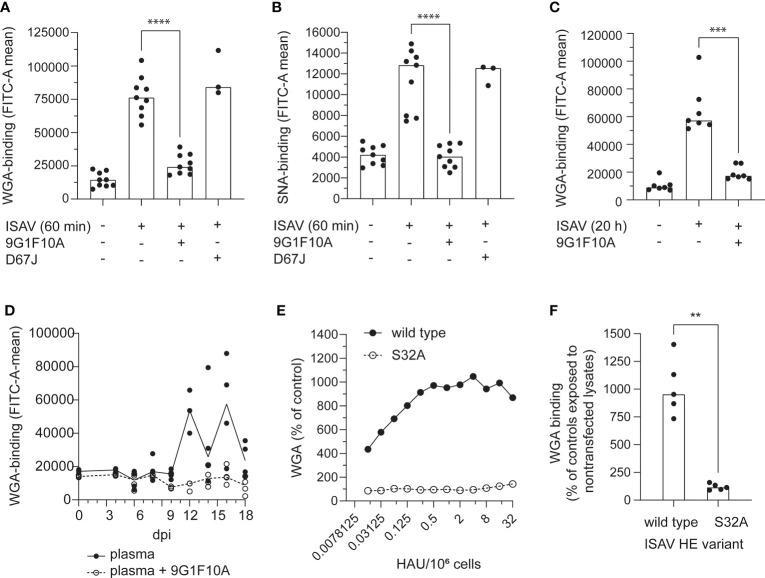
The pruning of erythrocyte surface is mediated by the ISAV esterase. **(A–C)** Virus supernatants were pre-incubated with antibodies targeting HE (9G1F10A) or the influenza A virus nucleoprotein (D67J) as a negative control (both: 5 µg/mL, 30 min, RT) before inoculation with erythrocytes (**A-B**: 10^6^ TCID_50_ per 20 million cells, 60 min; **C**: 10^5^ TCID_50_ per 20 million cells, 20 hours). Flow cytometry was used to evaluate **(A, C)** WGA-binding and **(B)** SNA-binding. **(D)** Plasma from infected fish harvested at the indicated time points was added to erythrocytes (10 µL/10^6^ cells) either alone (black dots and line) or pre-incubated with the antibody 9G1F10A (open circles, stippled line). Samples were incubated overnight before quantification of WGA-binding by flow cytometry. Data points show cells incubated with plasma from individual fish (4 per time point). The lines connect medians. **(E, F)** WGA-binding to erythrocytes exposed to wild type or esterase-silenced (S32A) HE at 1 HAU per 10^6^ cells for 20 hours. **(A-D, F)** Each data point represents blood cells isolated from one fish. Bars and line show median values. **p < 0.01, ***p < 0.001, ****p < 0.0001, Mann Whitney U.

A monoclonal antibody (mouse IgG_1_ clone 9G1F10A) that inhibited cellular ISAV attachment and hemagglutination (**Figure S2**) prevented the increase in WGA and SNA binding (**Figures 5A–C**). Similarly, the increase in WGA-binding observed when erythrocytes were incubated with plasma from infected fish, was strongly reduced when the plasma samples were pre-incubated with this neutralizing antibody (**Figure 5D**). Finally, incubating erythrocytes with recombinant HE showed that HE was sufficient to induce an increase in WGA-binding (**Figures 5E, F**). However, when the HE esterase activity was silenced by alanine mutation of its catalytic serine (S32) (34), the HE-mediated increase in WGA was abolished (**Figures 5E, F**).

Together, these findings suggest that ISAV-exposure renders erythrocyte sialic acids more available to lectin binding; that the attachment of ISAV to the cellular surface is required for this effect to take place; and that the ISAV-induced surface modulation at least in part is mediated by the hydrolytic activity of the viral RDE.

Our collected results support the conclusion that the ISAV RDE extensively removes 4-*O*-acetylated sialic acids from target cell surfaces in infected Atlantic salmon and suggest that this activity has potential to influence interactions with endogenous lectins.

## Discussion

We recently discovered that the ISAV receptor disappears from the vascular surface of ISAV-infected Atlantic salmon (23). Here, we confirm and expand on that observation, by showing that circulating erythrocytes in infected fish also lose the ability to bind new ISAV particles, and that the viral esterase has a key role in this process. Our findings support a model where the HE first attaches to cell surface 4-*O*-acetylated sialic acids and then causes extensive hydrolysis of adjacent non-bound 4-*O*-acetylated sialic acids. The RDE-mediated loss of cell surface 4-*O*-acetylated sialic acids limits further ISAV binding and makes cell surface sialic acids more available to lectins, but does not affect already-bound ISAV particles.

Despite being nucleated, and in contrast to endothelial cells (21), only a minor fraction of Atlantic salmon erythrocytes supports ISAV protein production (24). Our findings therefore illustrate that the loss of viral receptor does not depend on viral replication and suggest that the cell surface is modulated at an early stage of the infectious cycle.

The loss of erythrocyte ISAV binding was attributed to the hydrolytic activity of HE: First, the effect was not associated with receptor saturation; suggesting that it was mediated by hydrolysis of 4-*O*-acetyl sialic acids, like in endothelial cells (23). Second, the loss of ISAV receptor from vascular endothelial cells and erythrocytes correlated in individual fish, indicating a common denominator. Third, exposing normal erythrocytes to plasma from infected fish reproduced the surface modulation, suggesting that this denominator was present in plasma. The plasma-induced surface modulation was inhibited by an antibody that prevented ISAV receptor engagement, strengthening the assumption that circulating ISAV particles were responsible for the effect, rather than secreted host esterases. ISAV-dependent surface modulation was also observed when erythrocytes from non-infected fish were exposed to supernatants from infected cells. Finally, exposure to recombinant HE was sufficient to induce the same surface modulation in normal erythrocytes, but only when the HE hydrolytic activity was preserved.

Even though erythrocytes from infected fish no longer supported the attachment of new virus particles, ISAV remained associated with erythrocytes throughout the trial, with only a small decline over the last four days. The antibody used to quantify erythrocyte-associated ISAV specifically binds the ISAV surface protein HE (35); hence, it will not detect virus proteins after their release to the cytoplasm. However, it is possible that pre-fusion virus particles in endosomes could account for part of the observed signal. Nevertheless, several studies have suggested that the dissociation of ISAV from Atlantic salmon erythrocytes is limited. The isolate used in the current study, Glesvaer/2/90, was the first ISAV isolate propagated (36) and has since been used in many of the key publications that characterize ISAV proteins, including HE (16–19, 34). The isolate efficiently hydrolyses both free and glycosidically bound 4-*O*-acetylated sialic acids (18), and both horse, rabbit, rainbow trout, cod, and crucian carp erythrocytes elute in the expected manner after Glesvaer/2/90-mediated agglutination (16, 17). Yet, the virus does not permit elution of agglutinated Atlantic salmon erythrocytes (16, 17). Interestingly, the same lack of elution is seen when Atlantic salmon erythrocytes are agglutinated with influenza C virus (17), which binds and hydrolyses 9-*O*-acetylated sialic acids (37, 38). This suggests that the lack of virus dissociation might be due to specific properties of Atlantic salmon erythrocytes.

The discrepancy between hydrolytic activity and virus dissociation indicates that the release of ISAV from the cell surface depends more on the on-off rate of the initial receptor engagement than the viral esterase hydrolytic activity. In other words, the ISAV esterase appears to limit the initiation of new attachments, rather than inducing the dissociation of already-bound ISAV particles. Nevertheless, the hydrolysis of adjacent receptors will reduce the local receptor density around the site initially bound by the virus, limiting the possibility to reattach to the cellular surface after release of the initial attachment (3, 4).

The erythrocyte surface modulation in infected fish that abrogated further ISAV attachment also increased the binding of the plant lectin WGA. The hydroxyl-group of sialic acid carbon 4 contributes to determining the specificity of WGA (39). Also, the binding of WGA to a range of α2,3-linked sialic acids is inhibited by 4-*O*-acetylation (32). It is therefore not surprising that the ability to bind WGA increases when the ISAV receptor is lost. The effect on SNA-binding was less expected. SNA is highly specific to α2,6-linked sialic acids, and its binding is not inhibited by 9-*O*-acetylation (32). While we have not identified any previous studies of how 4-*O*-acetylation influences SNA-binding, our findings suggest that this modulation could limit interactions between SNA and its ligand on Atlantic salmon erythrocytes, presumably α2,6-linked sialic acids.

In addition to increasing the knowledge of how ISAV interacts with target cell surfaces in the early phase of the infectious cycle, our observations raise two important questions:

*First, does the RDE-induced cell surface-modulation influence biological functions in the host?* In viral infection, it is essential for a successful outcome that the immune response is powerful enough to eliminate the viral infection, yet controlled and specific enough to avoid unsustainable collateral damage to the host. In addition to being exploited by numerous microbes for attachment (2), sialic acids regulate host tolerance and immune activation by interacting with siglec-expressing immune cells (12–14, 40). These interactions can be modulated by sialic acid *O*-acetylation (41–43). Sialic acid de-acetylation, which we have identified on erythrocyte and vascular surfaces in ISAV-infected fish, typically enhances siglec interactions (41), and the biological end-result will depend on the specific siglec and cell type involved. ISA is characterized by a severe reduction in the number of circulating erythrocytes and circulatory collapse, but surprisingly low levels of perivascular inflammation, despite a strong vascular endothelial expression of viral proteins (20, 21). Amongst several interesting observations that may be relevant to ISA pathogenesis is that loss of 9-*O*-acetylation enhanced interactions between murine erythroid leukemia cells and siglec-1 and promoted erythroid cell retention in the spleen and liver (29). Furthermore, loss of *O*-acetylated sialic acids promoted the activation of natural killer cell inhibitory siglecs and protected human cancer cell lines from cytotoxicity (42). Atlantic salmon siglecs and other sialic acid-binding molecules remain poorly characterized. Hence, it is difficult to predict the effects of the ISAV RDE-induced loss of vascular and erythrocyte 4-*O*-acetylated sialic acids. Nevertheless, in the light of ISA pathogenesis, it is reasonable to ask if this virus-mediated cell surface modulation could play a role in the premature removal of erythrocytes from circulation or a possible inhibition of cytotoxic immune responses in infectious salmon anemia.

*Second, do other viral RDEs modulate target cell surfaces to a similar extent?* A range of viruses targets different host sialic acid derivatives as highly specific attachment receptors, and many of these also express a RDE (2). The influenza A virus neuraminidase promotes viral fitness (6, 44), but little is known about its direct effects on host cells during infection. The same is true for other viral RDEs. Evidence for destruction of the human erythrocyte influenza virus receptor was provided in hemagglutination reactions more than 70 years ago (45). Still, we have not identified any studies that address possible modulations of erythrocyte surfaces in influenza virus-infected hostss. Two studies have documented loss of host sialic acids in lung tissues of influenza A virus (H1N1, H1N2, and H4N6)- and highly pathogenic avian influenza A virus (HPAIV) (H5N1)-infected animals, as well as *ex vivo* HPAIV (H5N1)-infected human lung biopsies (46, 47). These studies support the assumption that influenza A virus, similar to ISAV, modulates target cell surfaces during infection. Interestingly, in contrast to the ISAV esterase that appears to make host cell surfaces more available to lectins that target de-acetylated sialic acids, the influenza A virus neuraminidase removes the virus-targeted sialic acid from the cell surface (48). Hence, it is possible that RDE activity could reduce interactions with immune-modulatory lectins and contribute to the dysregulated inflammation characteristic of severe human influenza (49, 50). While most influenza A strains rarely cause viraemia in humans, HPAIV (H5N1) is an exception (51). Considering the zoonotic and even pandemic potential of HPAIV (52), we suggest that the consequences of a widespread loss of virus-targeted sialic acids in the context of this infection should be addressed by future studies.

## Materials and methods

### Fish and experimental infection

Atlantic salmon (AquaGen, Trondheim, Norway) were hatched, reared, and kept at the aquaculture research station: Center for Sustainable Aquaculture (Norwegian University of Life Sciences [NMBU], Ås, Norway). Fish were maintained on a 24 hour light photoperiod in circular tanks in a temperature-controlled (14 ± 1°C) fresh water recirculatory aquaculture system and fed a standard salmon diet in excess, using automatic belt feeders. Blood for use in *ex vivo* experiments was collected in heparinized containers by terminal blood sampling from the caudal veins of fish (body weight 100-300 g) anesthetized by Tricaine methanesulfonate immersion (100-200 mg/L). Prior to the infection trial, the relevant fish group was tested by qPCR and found negative for infectious salmon anemia virus (gills/heart/kidney), salmon pox gill virus (gills), infectious pancreatic necrosis virus, piscine rheovirus-1, piscine myocarditis virus, and salmonid alphavirus (all heart/kidney) by the diagnostic services at the Norwegian Veterinary Institute.

A total of 73 fish (median body weight 115 g) were transferred to the NMBU infection Aquarium (Ås, Norway) for use in the infection trial. The unit uses a fresh water flow-through aquaculture system at 12°C. After acclimatization, one group (n=47) was infected by 2-hour immersion in water containing the high-virulent Norwegian ISAV isolate NO/Glesvaer/2/90 (36) (10^3.75^ TCID_50_/mL), as previously described (53). This protocol reliably infects all fish in a synchronized manner, but the severity of disease varies between trials (53, 54). Infected fish were sampled 4, 6, 8, 10, 12, 14, 16, and 18 dpi (n=4-6 fish per time point). A group of non-infected fish from the same batch (n=26) served as controls and was sampled 0, 12, 14, 16, and 18 dpi (n=4-6 fish per time point). At sampling, fish were anesthetized by immersion in benzocaine (40 mg/L), weighed and measured, and blood was collected from the caudal vein into heparinized containers. After blood sampling, the exterior of the fish was examined, fish were killed by cervical sectioning, the midline was incised, and organs were inspected and harvested. Hematocrits were measured within 90 min after sampling, by measuring the cell fraction of centrifuged blood (75 mm capillary tubes, 1200 rpm, 3 min, room temperature [RT]).

### Cells

Atlantic salmon kidney (ASK) cells (55) were maintained in L-15 medium (Lonza) supplemented with fetal bovine serum (FBS, 10%, Lonza), L-glutamine (Lonza, 4mM), and penicillin/streptomycin/amphotericin (Lonza, 1%) or gentamicin (50 µg/mL). The cells were cultured in closed cap tissue culture flasks at 20°C and were split 1:2 every 14 days.

Erythrocytes for *ex vivo* experiments were isolated from heparinized blood from non-infected Atlantic salmon by density gradient centrifugation. Briefly, 0.25 mL blood was diluted in 5 mL phosphate-buffered isotonic saline (PBS), layered on top of 7.5 mL 51% Percoll Plus (Sigma-Aldrich), and centrifuged without breaks (500x *g*, 20 min, 10°C). The erythrocyte pellets were extensively washed in PBS and resuspended 2 × 10^7^ cells/mL in the same culture medium as the ASK cells. Erythrocyte suspensions were maintained in 6-well cell culture plates on a microplate shaker (IKA-Werke, 150-200 rpm, 15°C) for up to 10 days. ISAV exposure was performed by resuspending erythrocytes in medium containing ISAV, plasma from infected fish, or recombinant HE (serial dilutions), followed by incubation in 24- or 48-well cell culture plates on a microplate shaker. Where relevant, ISAV or plasma from infected fish was pre-incubated with a mouse IgG_1_ targeting ISAV HE (clone 9G1F10A, generated by Knut Falk in the late 1990s) or mouse IgG_2a_ targeting influenza virus NP (clone D67J, Thermo Fisher Scientific) (both: 5 µg/mL, 30 min, RT).

### Virus and titer determination

ISAV was propagated in ASK cells at 15°C, and supernatants were harvested when cytopathic effects were close to complete, 14-28 dpi. Infective titers were determined by inoculating serial dilutions of supernatants or blood (10 µL heparinized blood was added to 500 µL L-15 medium and kept at -80°C until titration) in quadruplicate wells of ASK cells cultured in 96-well microtiter plates. Acetone-fixed cells were incubated with IgG_1_ against the ISAV nucleoprotein (P10, Aquatic Diagnostics Ltd, 0.4 µg/mL, 60 min, RT), washed (PBS × 3), and incubated with Alexa488-labelled goat anti-mouse IgG (A11001, Thermo Fisher Scientific, 5 µg/mL, 45 min, RT), and titers were calculated by the modified Kärber method, as previously described (17).

### RNA extraction and qPCR

20 µL heparinized blood was added to 400 µL MagNA Pure LC RNA Isolation Tissue Lysis buffer (Roche). Head kidney pieces (15-30 mm^3^) were collected in RNA later (Thermo Fisher Scientific), transferred to 500 µL MagNA Pure LC RNA Isolation Tissue Lysis buffer, and homogenized with 3-5 mm steel beads in a TissueLyser II (Qiagen, 24 Hz, 2 × 3 min). 350 µL lysed blood or 200 µL lysed head kidney were transferred to a Magna Pure 96 Processing Cartridge (Roche), and the total volume of head kidney samples was adjusted to 350 µL by adding MagNA Pure LC RNA Isolation Tissue Lysis buffer. RNA was extracted on a MagNA Pure 96 instrument (Roche) with the MagNA Pure 96 Cellular RNA Large Volume Kit (Roche), using the RNA tissue FF standard cellular RNA protocol with an elution volume of 50 µL per sample. RNA yield and purity was determined by a Multiskan Sky spectrophotometer (Thermo Fisher Scientific). The QuantiTect reverse transcription kit (Qiagen) was used for cDNA synthesis. Real time PCR was carried out with the TaqMan fast Advanced kit (Applied Biosystems) using the following protocol: TaqMan Fast advanced master mix (1x), TaqMan assay probe (0.2 µM), forward and reverse primers (0.5 µM), and cDNA template (0.5 ng/µl) final concentrations. Thermocycling was performed on a CFX384 Bio-Rad) and CFX-manager (software version 3.1, Bio-rad) under the following conditions: 2 min at 50 °C, 20 seconds at 95 °C, 40 cycles of 3 seconds each at 95 °C, and 30 seconds at 60 °C. Standard curves of a synthetic DNA fragment containing the relevant target sequences (Integrated DNA Technologies) were used for calculation of copy number per µg RNA. Sequences for primer/probes/DNA: ISAV segment 8: FW 5′-CGAAAGCCCTGGAAACTTTAGA-3′; REV 5′-GATGCCGGAAGTCGATGAACT-3′; probe FAM-aaggccatcgtcgct-NFQ. ELF-1α (for normalization): FW 5′-GGCTGGTTCAAGGGATGGA-3′; REV 5′-CAGAGTCACACCATTGGCGTTA-3′; probe FAM-tcgagcgtaaggatg-NFQ. DNA fragment: CATGCTGGAGGCTAGCGCCAACATGGGCTGGTTCAAGGGATGGAAGGTCGAGCGTAAGGATGGTAACGCCAATGGTGTGACTCTGCTGGAAGCCCTGGACGATGCAGATGTATGCTCTAGGAGCGAGTTCGAAAGCCCTGGAAACTTTAGAAAAGGCCATCGTCGCTGCAGTTCATCGACTTCCGGCATCCTGCTCGACAGAGAAGATGGTGCCAGGGTTGTATCCATGGTTGAAATGGACAGAGACGGCGTATCATTCATCTACGAGAAGCCTAGCATCTACCATAGTGATGGGTGCACTGGGACAGCATCGAGGGTCTGGAGACGGGATCACAATGAGAGAGCTGGAGTTGAGCTTAGGGCTGGACTTCACTTCAGAA.

### Flow cytometry

100 µL heparinized blood from each fish in the infection trial was collected in PBS, washed, and fixed (3% paraformaldehyde, 10 min, RT) before staining (RT). Erythrocytes from *ex vivo* experiments were stained live (4°C). Cells were labelled with Alexa488-labelled WGA (1 µg/mL, Thermo Fisher Scientific, 60 min), FITC-labelled SNA (10 µg/mL, Thermo Fisher Scientific, 60 min), or mouse IgG_1_ specific to the ISAV hemagglutinin esterase (clone 3H6F8 (35), hybridoma supernatants diluted 1:10 for fixed and 1:100 for live cells, 60 min), washed (PBS × 3), and (for detection of ISAV only) incubated with Alexa488-labelled goat anti-mouse IgG (A11001, Thermo Fisher Scientific, 5 µg/mL, 45 min). Signal was detected by a NovoCyte flow cytometer (Agilent), recording 25 000 events per sample.

### Immunostaining of blood smears

Blood smears were made and fixed (80% acetone, 15 min, RT) within 120 min of sampling, dried, and stored at -80°C until staining. Thawed and dried smears were incubated with 1x clear milk block (Thermo Fisher Scientific, 30 min, RT), mouse IgG_1_ specific to the ISAV HE (clone 3H6F8 (35), hybridoma supernatant diluted 1:100, 60 min, RT), washed (PBS × 3), and incubated with Alexa488-labelled goat anti-mouse IgG (A11001, Thermo Fisher Scientific, 5 µg/mL) together with Alexa594-labelled phalloidin (Thermo Fisher Scientific, 2 units/mL, 45 min, RT). Nuclei were counterstained by Hoechst 33342 (Thermo Fisher Scientific, 2 µg/mL, 5 min, RT), and slides were mounted in ProLong Gold antifade mountant (Thermo Fisher Scientific). Wide-field microscopy was performed by a Nikon Ti-2e inverted microscope, using a Plan Apo lambda DIC N2 63x oil objective (NA 1.4). The percentage of ISAV-positive cells in individual fish was quantified in ImageJ (version 1.53c) (56): Total cell numbers were determined using the *Analyze particles* function on thresholded images generated from the Hoechst channel. Next, the number of ISAV-positive cells were counted manually in the channel containing signal from the HE-staining, assessing a minimum of 99 cells per fish.

### Histology

An organ panel including heart, spleen, and head kidney was collected from each fish, fixed in 10% formalin for at least 24 hours, dehydrated, and embedded in paraffin. Thin tissue sections were heat treated (60-70°C, 20 min), deparaffinized, and either stained with hematoxylin & eosin for histological evaluation or subjected to a virus binding assay, as described below.

### Preparation and blotting of membrane-enriched lysates

Cell pellets were prepared from full blood or density-purified erythrocytes and stored at -80°C before preparation of membrane-enriched fractions as previously described (24). Briefly, 100 µL cell pellets were lysed by 1:10 dilution in ice cold water with 1% protease inhibitor (P8340, Sigma-Aldrich, 10 min, on ice). The cells were homogenized with a tight-fitting Dounce homogenization (20 strokes), 1000 µL buffer A (75 mM Tris pH 7.5, 12.5 mM MgCl, 15 mM EDTA) was added, and the homogenization was repeated. To remove nuclei and organelles, the suspension was centrifuged (5000× *g*, 5 min), and the supernatant was collected on ice. The homogenization procedure was repeated three times with the cell pellet in buffer A diluted 1:1 with water, pooled supernatant fractions were centrifuged (40,000× *g*, 30 min), the membrane-enriched pellets were resuspended in 50 µL buffer B (20 mM Tris, 2 mM EDTA, pH 7.5), and samples were stored at -80°C. After thawing, 10 µL sample, 3.88 µL NuPage LDS sample buffer, and 1.55 µL NuPage sample reducing agent (both from Thermo Fisher Scientific) was mixed and homogenized by centrifugation in QiaShredder columns (Qiagen), heat-treated (10 min, 70°C), and loaded to gels (8 µL/well) for SDS-PAGE and Western blotting (NuPage Novex system, Thermo Fisher Scientific) to 0.45 µm nitrocellulose membranes (BioRad laboratories).

### Production of antigen for virus binding assays

Antigens for virus histochemistry and virus binding assays were prepared by collecting membrane fractions of infected ASK cells, as previously described (21). Briefly, cells in 75 cm^2^ tissue culture flasks were harvested when cytopathic effects were evident but most cells remained attached, typically 3-7 dpi. Cells were washed and scraped on ice, and cell pellets containing cellular-expressed viral membrane glycoproteins were washed, resuspended in 0.5 mL PBS, and subjected to three rounds of freeze-thawing. Hemagglutination titers were determined by incubating serial dilutions of antigen preparations with 1% Atlantic salmon erythrocyte suspensions in 96-well V-bottom microtiter plates.

### Virus binding assays

To map virus-binding sites in Atlantic salmon tissues and membrane-enriched cell fractions, we used virus antigen preparations as primary probes as previously described (21, 23, 24).

Deparaffinized formalin-fixed tissue sections were incubated with 100 µL ISAV antigen (512 HAU/mL, 60 min, RT), washed (PBS × 3), quenched with peroxidase block (5 min, RT), treated with blocking buffer (Background sniper, Biocare Medical, 30 min, RT), incubated with mouse IgG_1_ specific to ISAV HE (clone 3H6F8 (35), hybridoma supernatants diluted 1:100, 60 min, RT), washed (PBS × 3), and signal was visualized by the MACH2 HRP polymer-DAB (Biocare Medical) system, following manufacturer’s instructions. Signal was analyzed as described in the section *Quantification of virus histochemistry signal*.

Blotted membrane-enriched cell lysates (prepared as described above) were stained for total protein using Revert700 Total Protein Stain as recommended by the manufacturer (Licor Biosciences), treated with dry milk (5% in Tris-buffered saline [TBS], 60 min, RT), washed (TBS 0.1% Tween [TBST] × 2), incubated with ISAV antigen (256 HAU/mL diluted in TBS, 60 min, RT), washed (TBST × 3), incubated with mouse IgG_1_ specific to ISAV HE (clone 3H6F8 (35), hybridoma supernatants diluted 1:250 in TBST with 5% dry milk, 60 min, RT), and washed (TBST × 3). Bound primary antibody was detected either by incubation with IRDye800-labelled goat anti-mouse IgG (Li-Cor Biosciences, 1:10,000 in TBST with 5% dry milk, 60 min, RT), or by incubation with HRP-conjugated horse anti-mouse IgG (Cell Signaling, 1:1000, 60 min, RT), wash (TBST x 3), and incubation with Super Signal West pico substrate (Thermo Fisher scientific). Chemiluminescent or fluorescent signal was visualized by an Azure imager C500 (Azure Biosystems). For quantification, fluorescent signal was normalized to total protein signal, using Azure spot software (Azure Biosystems, version 2.2.167).

### Quantification of virus histochemistry signal

Images of tissue sections subjected to virus binding assays were digitized using a Hamamatsu Nanozoomer digital slide scanner with 40x objective (114932 pixels per inch, 221 nm/pixel, JPEG compression at 80%). The virus histochemistry signal was analyzed using Visiopharm, v2022.2. For region of interest (= organ) detection and area measurement, a generic analysis protocol package using artificial intelligence (DeepLab V3) trained in our lab on collections of slides containing Atlantic salmon liver, heart, kidney, spleen and adipose tissue, was retrained on the full set of stained sections from the present study. Signal detection was performed at 20x magnification: The image pixel value was inverted, multiplied by 5, and combined with their red-green contrast values using a maximum pixel value filter. Pixels with resulting values above a threshold of 50 were considered positive. The total area covered by positive pixels per organ was divided by organ area to yield the fraction of positive signal relative to area of the organ in the section.

### Generation of recombinant ISAV HE

Codon optimized sequences of the open reading frame encoding ISAV HE, corresponding to isolate NO/Finnmark/NVI-70-1250/2020 (Genbank accession UGL76651.1), as well as a variant where serine 32 was mutated to alanine to abolish catalytic activity (S32A), was synthesized and inserted in the pcDNA3.1 (+) vector commercially and delivered transfection-ready (GeneCust). Monolayers of CHSE-214 cells were cultured until 90-100% confluent and detached by Trypsin EDTA (Lonza). 4 transfection reactions, each with 10^6^ cells and 10 µg plasmid DNA, were performed, using the Neon 100 µL Transfection System (Invitrogen, Waltham, MA, USA, three pulses at 1600 V and 10 ms width). Non-transfected cells were used as controls. The transfected cells were pooled and incubated 24 hours in antibiotic free medium, then another 24 hours in culture medium. Membrane fractions were collected as described for infected cells and previously (21).

### Statistics

Statistics were performed in Graph Pad Prism 8 for Windows 64-bits, v.8.4.3.

## Data availability statement

The original contributions presented in the study are included in the article/**Supplementary Material**. Further inquiries can be directed to the corresponding author.

## Ethics statement

Protocols for harvesting material from healthy fish and experimental infection and their implementation were approved prior to the studies by the Norwegian Food Safety Authority (FOTS24382, FOTS28403). All facilities were operated in compliance with Organization for Economic Co-operation and Development principles of Good Laboratory Practice and Guidelines to Good Manufacturing Practice issued by the European Commission.

## Author contributions

Conceptualization: JF, KF; Design, planning, and supervision: JF, AA, KF; Experimentation: JF, AA, FP, SW, IH, SS, KL, AS; Analysis and interpretation: JF, AA, FP, SW, AS; Initial draft and figures: JF. Revision of initial draft: All authors. Funding: KF. All authors contributed to the article and approved the submitted version.

## Supplementary material

Supplementary Table 1 Numericals and statistics used to generate the figures.

Supplementary Figure 1 Supplemental to **Figure 1** : **(A, B)** Manual scoring and **(C)** example micrograph of erythrophagocytosis in **(A)** head kidney and **(B, C)** spleen in hematoxylin and eosin-stained formalin-fixed, paraffin-embedded tissue sections of individual fish. Arrows point to examples of frequent erythrophagocytosis and pigmentation in spleen of an infected fish harvested 16 dpi, indicating breakdown of hemoglobin. **(D)** Viral RNA in head kidney was measured by qPCR targeting ISAV segment 8. Data points show values in individual fish, the line connects median values. **(E)** Gating strategy and examples of histograms showing HE-staining in PFA-fixed erythrocytes from non-infected (black) and infected (red) fish, harvested 12 dpi.

Supplementary Figure 2 Supplemental to **Figure 5**: **(A)** ISAV was pre-incubated with monoclonal antibodies as indicated (5 µg/mL, 30 min, RT), before inoculation with density-purified erythrocytes isolated from non-infected fish (10^6^ TCID_50_ per 2 ×10^7^ cells, 60 min) and quantification of HE by flow cytometry. Data points show measurements in cells from individual fish. **p<0.01, Kruskal-Wallis with Dunn’s multiple comparisons test (**Table S1**). **(B)** Hemagglutination inhibition assay testing the ability of 9G1F10A to inhibit ISAV-induced agglutination (4 HAU ISAV antigen and 10^6^ erythrocytes per well). The boxed area indicates the concentration range of the antibody that completely inhibited agglutination.

## References

[1] Lewis AL Chen X Schnaar RL Varki A. Sialic acids and other nonulosonic acids. In: Varki A Cummings RD Esko JD Stanley P Hart GW, editors. Essentials of glycobiology. Cold Spring Harbor (NY (2022). p. 185–204.

[2] Matrosovich M Herrler G Klenk HD. Sialic acid receptors of viruses. Top Curr Chem (2015) 367:1–28. doi: 10.1007/128_2013_466 23873408 PMC7120183

[3] Guo H Rabouw H Slomp A Dai M van der Vegt F van Lent JWM. Kinetic analysis of the influenza a virus HA/NA balance reveals contribution of NA to virus-receptor binding and NA-dependent rolling on receptor-containing surfaces. PLoS Pathog (2018) 14(8):e1007233. doi: 10.1371/journal.ppat.1007233 30102740 PMC6107293

[4] Sakai T Nishimura SI Naito T Saito M. Influenza a virus hemagglutinin and neuraminidase act as novel motile machinery. Sci Rep (2017) 7:45043. doi: 10.1038/srep45043 28344335 PMC5366856

[5] Strobl B Vlasak R. The receptor-destroying enzyme of influenza c virus is required for entry into target cells. Virology (1993) 192(2):679–82. doi: 10.1006/viro.1993.1087 8421907

[6] Matrosovich MN Matrosovich TY Gray T Roberts NA Klenk HD. Neuraminidase is important for the initiation of influenza virus infection in human airway epithelium. J Virol (2004) 78(22):12665–7. doi: 10.1128/JVI.78.22.12665-12667.2004 PMC525087 15507653

[7] Palese P Tobita K Ueda M Compans RW. Characterization of temperature sensitive influenza virus mutants defective in neuraminidase. Virology (1974) 61(2):397–410. doi: 10.1016/0042-6822(74)90276-1 4472498

[8] Horga MA Gusella GL Greengard O Poltoratskaia N Porotto M Moscona A. Mechanism of interference mediated by human parainfluenza virus type 3 infection. J Virol (2000) 74(24):11792–9. doi: 10.1128/jvi.74.24.11792-11799.2000 PMC112462 11090179

[9] Huang IC Li W Sui J Marasco W Choe H Farzan M. Influenza a virus neuraminidase limits viral superinfection. J Virol (2008) 82(10):4834–43. doi: 10.1128/JVI.00079-08 PMC2346733 18321971

[10] Goto H Ohta K Matsumoto Y Yumine N Nishio M. Evidence that receptor destruction by the Sendai virus hemagglutinin-neuraminidase protein is responsible for homologous interference. J Virol (2016) 90(17):7640–6. doi: 10.1128/JVI.01087-16 PMC4988132 27279623

[11] Morrison TG McGinnes LW. Avian cells expressing the Newcastle disease virus hemagglutinin-neuraminidase protein are resistant to Newcastle disease virus infection. Virology (1989) 171(1):10–7. doi: 10.1016/0042-6822(89)90505-9 2545025

[12] Saini P Adeniji OS Abdel-Mohsen M. Inhibitory siglec-sialic acid interactions in balancing immunological activation and tolerance during viral infections. EBioMedicine (2022) 86:104354. doi: 10.1016/j.ebiom.2022.104354 36371982 PMC9663867

[13] Laubli H Varki A. Sialic acid-binding immunoglobulin-like lectins (Siglecs) detect self-associated molecular patterns to regulate immune responses. Cell Mol Life Sci (2020) 77(4):593–605. doi: 10.1007/s00018-019-03288-x 31485715 PMC7942692

[14] Macauley MS Crocker PR Paulson JC. Siglec-mediated regulation of immune cell function in disease. Nat Rev Immunol (2014) 14(10):653–66. doi: 10.1038/nri3737 PMC4191907 25234143

[15] Mjaaland S Rimstad E Falk K Dannevig BH. Genomic characterization of the virus causing infectious salmon anemia in Atlantic salmon (*Salmo salar* l.): an orthomyxo-like virus in a teleost. J Virol (1997) 71(10):7681–6. doi: 10.1128/JVI.71.10.7681-7686.1997 PMC192118 9311851

[16] Falk K Aspehaug V Vlasak R Endresen C. Identification and characterization of viral structural proteins of infectious salmon anemia virus. J Virol (2004) 78(6):3063–71. doi: 10.1128/jvi.78.6.3063-3071.2004 PMC353767 14990725

[17] Falk K Namork E Rimstad E Mjaaland S Dannevig BH. Characterization of infectious salmon anemia virus, an orthomyxo-like virus isolated from Atlantic salmon (*Salmo salar* l. ) J Virol (1997) 71(12):9016–23. doi: 10.1128/JVI.71.12.9016-9023.1997 PMC230202 9371558

[18] Hellebo A Vilas U Falk K Vlasak R. Infectious salmon anemia virus specifically binds to and hydrolyzes 4-*O*-acetylated sialic acids. J Virol (2004) 78(6):3055–62. doi: 10.1128/jvi.78.6.3055-3062.2004 PMC353765 14990724

[19] Cook JD Sultana A Lee JE. Structure of the infectious salmon anemia virus receptor complex illustrates a unique binding strategy for attachment. Proc Natl Acad Sci U S A (2017) 114(14):E2929–E36. doi: 10.1073/pnas.1617993114 PMC5389325 28320973

[20] Falk K Aamelfot M. Infectious salmon anaemia. In: Fish viruses and bacteria: pathobiology and protection (2017). p. 68–78. CABI (Oxfordhire, UK).

[21] Aamelfot M Dale OB Weli SC Koppang EO Falk K. Expression of the infectious salmon anemia virus receptor on Atlantic salmon endothelial cells correlates with the cell tropism of the virus. J Virol (2012) 86(19):10571–8. doi: 10.1128/JVI.00047-12 PMC3457268 22811536

[22] Koren CWR Nylund A. Morphology and morphogenesis of infectious salmon anaemia virus replicating in the endothelium of Atlantic salmon salmo salar. Dis Aquat Organisms (1997) 29:99–109. doi: 10.3354/dao029099

[23] Aamelfot M Fosse JH Viljugrein H Ploss FB Benestad SL McBeath A. Destruction of the vascular viral receptor in infectious salmon anaemia provides *in vivo* evidence of homologous attachment interference. PLoS Pathog (2022) 18(10):e1010905. doi: 10.1371/journal.ppat.1010905 36240255 PMC9621750

[24] Fosse JH Aamelfot M Sønstevold T Weli SC Vendramin N Petersen PE. Salmon erythrocytes sequester active virus particles in infectious salmon anaemia. Viruses (2022) 14. doi: 10.3390/v14020310 PMC8879071 35215905

[25] Thorud KE. Infectious salmon anaemia: transmission trial: haematological, clinical, chemical and morphological investigations. Oslo: Norwegian Veterinary College (1991).

[26] Jancik J Schauer R. Sialic acid–a determinant of the life-time of rabbit erythrocytes. Hoppe Seylers Z Physiol Chem (1974) 355(4):395–400. doi: 10.1515/bchm2.1974.355.1.395 4435729

[27] Durocher JR Payne RC Conrad ME. Role of sialic acid in erythrocyte survival. Blood (1975) 45(1):11–20. doi: 10.1182/blood.V45.1.11.11 803103

[28] Bratosin D Mazurier J Debray H Lecocq M Boilly B Alonso C. Flow cytofluorimetric analysis of young and senescent human erythrocytes probed with lectins. evidence that sialic acids control their life span. Glycoconj J (1995) 12(3):258–67. doi: 10.1007/BF00731328 7496140

[29] Shi WX Chammas R Varki NM Powell L Varki A. Sialic acid 9-o-acetylation on murine erythroleukemia cells affects complement activation, binding to I-type lectins, and tissue homing. J Biol Chem (1996) 271(49):31526–32. doi: 10.1074/jbc.271.49.31526 8940168

[30] Sjoberg ER Powell LD Klein A Varki A. Natural ligands of the b cell adhesion molecule CD22 beta can be masked by 9-o-acetylation of sialic acids. J Cell Biol (1994) 126(2):549–62. doi: 10.1083/jcb.126.2.549 PMC2200033 8034751

[31] Cariappa A Takematsu H Liu H Diaz S Haider K Boboila C. B cell antigen receptor signal strength and peripheral b cell development are regulated by a 9-o-acetyl sialic acid esterase. J Exp Med (2009) 206(1):125–38. doi: 10.1084/jem.20081399 PMC2626685 19103880

[32] Srivastava S Verhagen A Sasmal A Wasik BR Diaz S Yu H. Development and applications of sialoglycan-recognizing probes (SGRPs) with defined specificities: exploring the dynamic mammalian sialoglycome. Glycobiology (2022) 32(12):1116–1136. doi: 10.1093/glycob/cwac050 PMC9680117 35926090

[33] Bornhofft KF Goldammer T Rebl A Galuska SP. Siglecs: a journey through the evolution of sialic acid-binding immunoglobulin-type lectins. Dev Comp Immunol (2018) 86:219–31. doi: 10.1016/j.dci.2018.05.008 29751010

[34] Muller A Markussen T Drablos F Gjoen T Jorgensen TO Solem ST. Structural and functional analysis of the hemagglutinin-esterase of infectious salmon anaemia virus. Virus Res (2010) 151(2):131–41. doi: 10.1016/j.virusres.2010.03.020 PMC7114507 20398710

[35] Falk K Namork E Dannevig BH. Characterization and applications of a monoclonal antibody against infectious salmon anaemia virus. Dis Aquat Organ (1998) 34(2):77–85. doi: 10.3354/dao034077 9828403

[36] Dannevig BH Falk K Namork E. Isolation of the causal virus of infectious salmon anaemia (ISA) in a long-term cell line from Atlantic salmon head kidney. J Gen Virol (1995) 76:1353–9. doi: 10.1099/0022-1317-76-6-1353 7782764

[37] Rogers GN Herrler G Paulson JC Klenk HD. Influenza c virus uses 9-O-acetyl-N-acetylneuraminic acid as a high affinity receptor determinant for attachment to cells. J Biol Chem (1986) 261(13):5947–51. doi: 10.1016/s0021-9258(17)38475-2 3700379

[38] Herrler G Rott R Klenk HD Müller HP Shukla AK Schauer R. The receptor-destroying enzyme of influenza c virus is neuraminate-o-acetylesterase. EMBO J (1985) 4(6):1503–6. doi: 10.1002/j.1460-2075.1985.tb03809.x PMC554374 2411539

[39] Sharon N. Lectin-carbohydrate complexes of plants and animals: an atomic view. Trends Biochem Sci (1993) 18(6):221–6. doi: 10.1016/0968-0004(93)90193-q 8346557

[40] Lubbers J Rodriguez E van Kooyk Y. Modulation of immune tolerance *via* siglec-sialic acid interactions. Front Immunol (2018) 9:2807. doi: 10.3389/fimmu.2018.02807 30581432 PMC6293876

[41] Visser EA Moons SJ Timmermans S de Jong H Boltje TJ Bull C. Sialic acid *O*-acetylation: from biosynthesis to roles in health and disease. J Biol Chem (2021) 297(2):100906. doi: 10.1016/j.jbc.2021.100906 34157283 PMC8319020

[42] Grabenstein S Barnard KN Anim M Armoo A Weichert WS Bertozzi CR. Deacetylated sialic acids modulates immune mediated cytotoxicity *via* the sialic acid-siglec pathway. Glycobiology (2021) 31(10):1279–94. doi: 10.1093/glycob/cwab068 34192335

[43] Schauer R Srinivasan GV Wipfler D Kniep B Schwartz-Albiez R. O-Acetylated sialic acids and their role in immune defense. Adv Exp Med Biol (2011) 705:525–48. doi: 10.1007/978-1-4419-7877-6_28 PMC7123180 21618128

[44] Yang J Liu S Du L Jiang S. A new role of neuraminidase (NA) in the influenza virus life cycle: implication for developing NA inhibitors with novel mechanism of action. Rev Med Virol (2016) 26(4):242–50. doi: 10.1002/rmv.1879 PMC7169148 27061123

[45] Hirst GK. Adsorption of influenza hemagglutinins and virus by red blood cells. J Exp Med (1942) 76(2):195–209. doi: 10.1084/jem.76.2.195 19871229 PMC2135226

[46] van Riel D Leijten LM Kochs G Osterhaus A Kuiken T. Decrease of virus receptors during highly pathogenic H5N1 virus infection in humans and other mammals. Am J Pathol (2013) 183(5):1382–9. doi: 10.1016/j.ajpath.2013.07.004 23993779

[47] Trebbien R Larsen LE Viuff BM. Distribution of sialic acid receptors and influenza a virus of avian and swine origin in experimentally infected pigs. Virol J (2011) 8:434. doi: 10.1186/1743-422X-8-434 21902821 PMC3177912

[48] Lai JCC Karunarathna H Wong HH Peiris JSM Nicholls JM. Neuraminidase activity and specificity of influenza a virus are influenced by haemagglutinin-receptor binding. Emerg Microbes Infect (2019) 8(1):327–38. doi: 10.1080/22221751.2019.1581034 PMC6455212 30866786

[49] Kalil AC Thomas PG. Influenza virus-related critical illness: pathophysiology and epidemiology. Crit Care (2019) 23(1):258. doi: 10.1186/s13054-019-2539-x 31324202 PMC6642581

[50] Liu Q Liu DY Yang ZQ. Characteristics of human infection with avian influenza viruses and development of new antiviral agents. Acta Pharmacol Sin (2013) 34(10):1257–69. doi: 10.1038/aps.2013.121 PMC3791557 24096642

[51] Kuiken T van den Brand J van Riel D Pantin-Jackwood M Swayne DE. Comparative pathology of select agent influenza a virus infections. Vet Pathol (2010) 47(5):893–914. doi: 10.1177/0300985810378651 20682805

[52] Yamaji R Saad MD Davis CT Swayne DE Wang D Wong FYK. Pandemic potential of highly pathogenic avian influenza clade 2.3.4.4 A(H5) viruses. Rev Med Virol (2020) 30(3):e2099. doi: 10.1002/rmv.2099 32135031 PMC9285678

[53] McBeath A Aamelfot M Christiansen DH Matejusova I Markussen T Kaldhusdal M. Immersion challenge with low and highly virulent infectious salmon anaemia virus reveals different pathogenesis in Atlantic salmon, *Salmo salar* l. J Fish Dis (2015) 38(1):3–15. doi: 10.1111/jfd.12253 24820820

[54] Fosse JH Haraldsen G Falk K Edelmann R. Endothelial cells in emerging viral infections. Front Cardiovasc Med (2021) 8:619690. doi: 10.3389/fcvm.2021.619690 33718448 PMC7943456

[55] Devold M Krossoy B Aspehaug V Nylund A. Use of RT-PCR for diagnosis of infectious salmon anaemia virus (ISAV) in carrier sea trout *Salmo trutta* after experimental infection. Dis Aquat Organ (2000) 40(1):9–18. doi: 10.3354/dao040009 10785858

[56] Schindelin J Arganda-Carreras I Frise E Kaynig V Longair M Pietzsch T. Fiji: An open-source platform for biological-image analysis. Nat Methods (2012) 9(7):676–82. doi: 10.1038/nmeth.2019 PMC3855844 22743772

