# Supplementary figures and images for: The infectious salmon anemia virus esterase prunes erythrocyte surfaces in infected Atlantic salmon and exposes terminal sialic acids to lectin recognition
Source: Front Immunol. 2023 Apr 25;14:1158077. doi: 10.3389/fimmu.2023.1158077 (PMC10167051; doi:10.3389/fimmu.2023.1158077)

**A**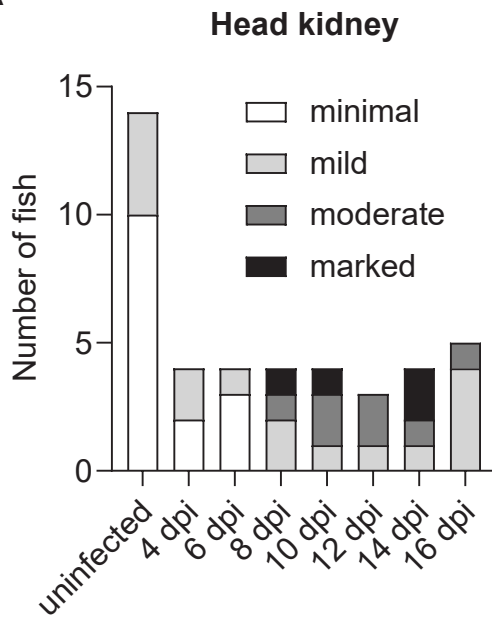**B**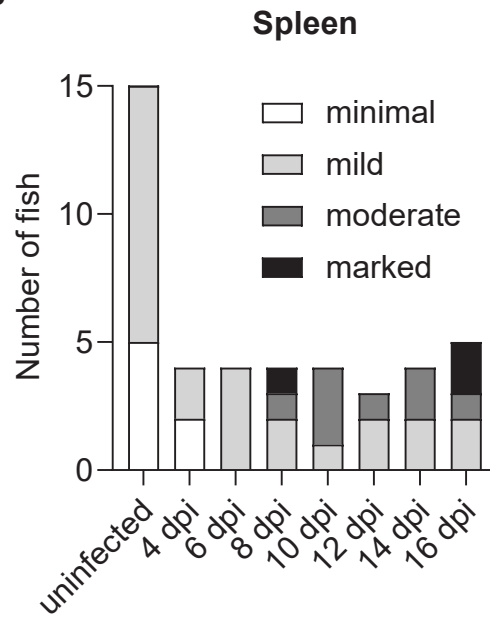**C**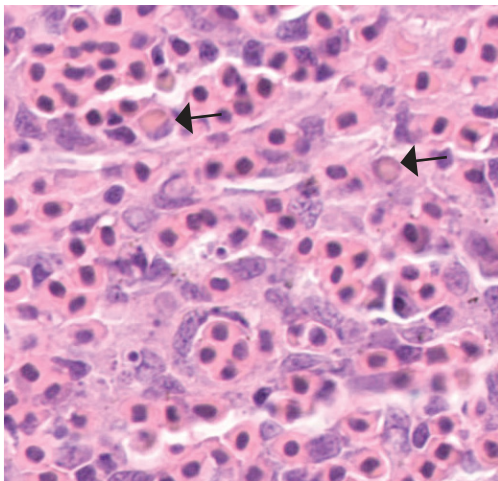

Spleen of infected fish, 16 dpi

**D**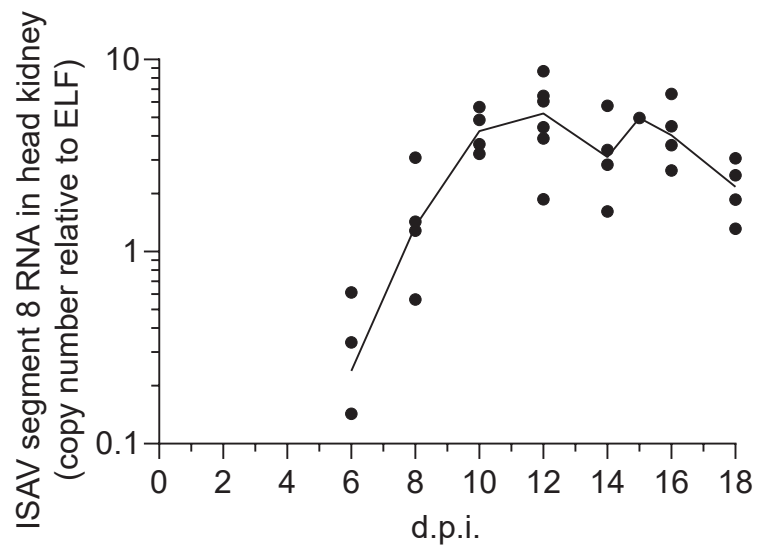**E**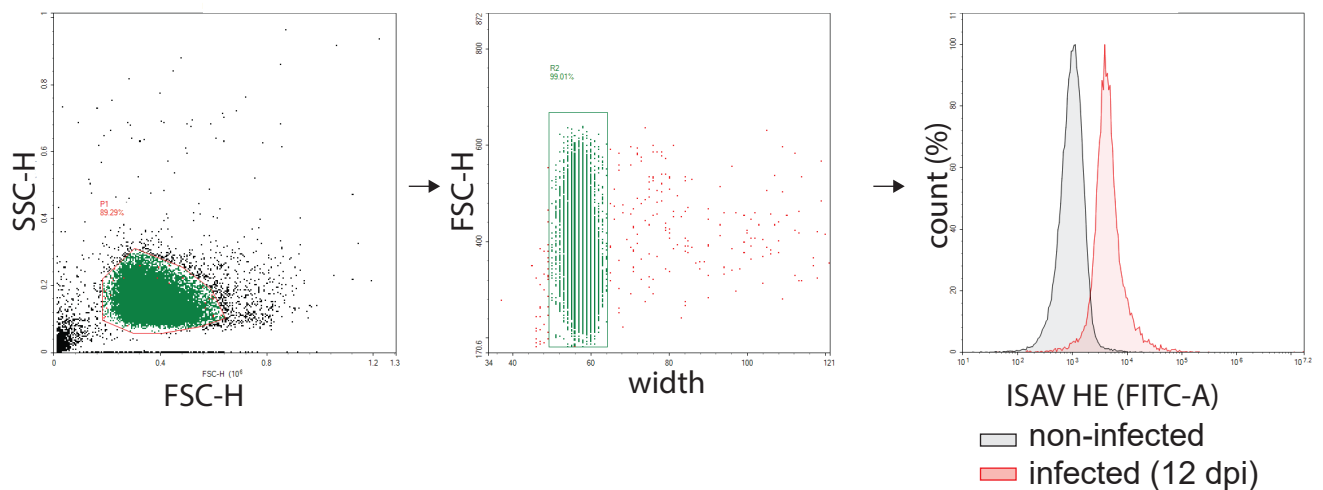

Supplement: Supplementary Figure 1 — Supplemental to Figure 1 : (A, B) Manual scoring and (C) example micrograph of erythrophagocytosis in (A) head kidney and (B, C) spleen in hematoxylin and eosin-stained formalin-fixed, paraffin-embedded tissue sections of individual fish. Arrows point to examples of frequent erythrophagocytosis and pigmentation in spleen of an infected fish harvested 16 dpi, indicating breakdown of hemoglobin. (D) Viral RNA in head kidney was measured by qPCR targeting ISAV segment 8. Data points show values in individual fish, the line connects median values. (E) Gating strategy and examples of histograms showing HE-staining in PFA-fixed erythrocytes from non-infected (black) and infected (red) fish, harvested 12 dpi. [file Image_1.pdf]

**A**

ISAV HE signal (FITC-A mean)

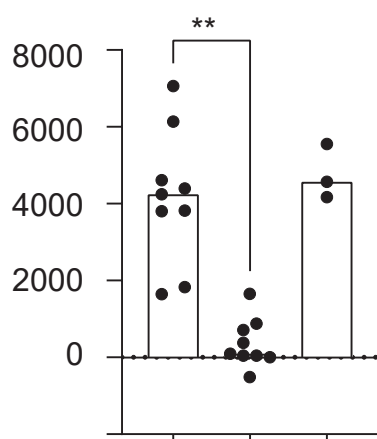

ISAV (60 min)

+

9G1F10A

-

D67J

-

+

**B**mAb conc ( $\mu\text{g/mL}$ )

5

2.5

1.25

0.625

0.313

0.156

9G1F10A

no antibody

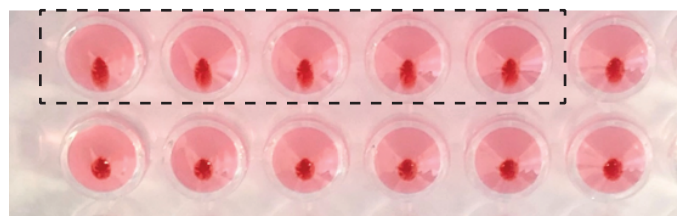

Supplement: Supplementary Figure 2 — Supplemental to Figure 5 : (A) ISAV was pre-incubated with monoclonal antibodies as indicated (5 µg/mL, 30 min, RT), before inoculation with density-purified erythrocytes isolated from non-infected fish (106 TCID50 per 2 ×10^7^ cells, 60 min) and quantification of HE by flow cytometry. Data points show measurements in cells from individual fish. **p<0.01, Kruskal-Wallis with Dunn’s multiple comparisons test ( Table S1 ). (B) Hemagglutination inhibition assay testing the ability of 9G1F10A to inhibit ISAV-induced agglutination (4 HAU ISAV antigen and 106 erythrocytes per well). The boxed area indicates the concentration range of the antibody that completely inhibited agglutination. [file Image_2.pdf]
